# A neutrosophic explainable AI framework for modeling uncertainty in immersive stereotactic neurosurgical simulation

**DOI:** 10.3389/fneur.2026.1766089

**Published:** 2026-02-23

**Authors:** Jesus Rafael Hechavarria-Hernandez

**Affiliations:** Universidad Ecotec, Samborondón, Ecuador

**Keywords:** deep brain stimulation, explainable AI, neutrosophic logic, stereotactic neurosurgery, uncertainty modeling

## Abstract

The integration of Artificial Intelligence (AI) and Virtual Reality (VR) has transformed medical education; however, performance assessment in high-stakes fields such as stereotactic neurosurgery remains largely dependent on binary or threshold-based metrics. In procedures such as deep brain stimulation (DBS), where safety margins are below 2 mm, these approaches fail to capture indeterminate behaviors, including hesitation, micro-instability, and unstable trajectories, potentially leading to false-positive competence classifications. This study introduces a Neutrosophic Explainable AI (N-XAI) framework that models surgical performance through three independent dimensions: truth (competence), Indeterminacy (instability/ambiguity), and Falsity (error). Performance is represented in a two-dimensional precision–stability space and quantified using single-valued neutrosophic sets (SVNS). For theoretical validation, a synthetic dataset comprising 60 simulated surgical attempts distributed across three skill groups (expert, indeterminate, and novice) was generated. Neutrosophic competence scores were computed and analyzed using non-parametric statistical tests. The framework successfully differentiated the three groups and identified indeterminate, high-risk cases that achieved acceptable spatial accuracy but exhibited significant instability—patterns that conventional metrics fail to detect. The proposed N-XAI framework provides a mathematically grounded and interpretable approach for modeling uncertainty in immersive neurosurgical simulation. By explicitly accounting for indeterminacy, it enhances the diagnostic value of VR-based training systems and lays the groundwork for future validation in live stereotactic simulation environments.

## 1 Introduction

Virtual and extended reality (VR/XR) technologies have become essential tools in neurosurgical education and rehearsal. Over the past decade, multiple studies have demonstrated that immersive simulators can enhance spatial understanding, psychomotor skills, and preoperative planning in complex cranial and spinal procedures. Recent reviews confirm that VR and augmented reality are transforming not only preoperative visualization but also the intraoperative workflow and team coordination in the neurosurgical operating room. Additionally, simulation-based programs proved particularly valuable during the COVID-19 pandemic, when access to elective surgical cases was greatly limited. At the same time, forward-looking analyses suggest that VR will remain a key part of medical education in the coming decade, serving as a platform for scalable, data-driven training. Key component of medical education in the coming decade, acting as a platform for scalable, data-driven training (1, 2).

Despite these advances in realism and adoption, the evaluation components of most neurosurgical simulators remain relatively underdeveloped. Current systems generally depend on a limited set of performance metrics—such as task completion time, path length, or a geometric deviation threshold relative to a predefined target (3–5). These criteria are helpful but do not fully capture the subtle psychomotor behaviors that determine whether a trajectory is clinically safe. This limitation is especially clear in settings where access to cadaver labs and high-fidelity models is limited, including low- and middle-income environments (6), increasing the need to rely on reliable and interpretable metrics from VR-based training alone. Even in specialized training tools, such as intraventricular neuroendoscopy simulators, assessment often depends on global rating scales or broad threshold rules (7).

The need for more rigorous assessment is especially critical in functional and stereotactic neurosurgery. Deep brain stimulation (DBS) for Parkinson's disease and other movement disorders has demonstrated strong long-term benefits across several multicenter studies. However, these results heavily depend on the precision of electrode placement within small subcortical targets, such as the subthalamic nucleus (STN). Even millimeter deviations in lead position or errors in trajectory planning can significantly impact motor outcomes and increase complication rates, as highlighted by large MRI-guided and MRI-verified DBS series reporting correlations between targeting accuracy and clinical safety outcomes (8–10). These concerns around precision are underscored by clinical discussions advocating stringent consideration of risk, safety margins, and trajectory optimisation when applying stereotactic techniques in severe refractory conditions such as obsessive-compulsive disorder and depression (11). Contemporary efforts to optimize functional neurosurgical workflows—including efficiency, targeting accuracy, and procedure safety—further reinforce that improvements in surgical planning and execution are critical to maintaining high standards of care (12). As neuromodulation techniques expand and diversify, there is increasing recognition that surgical training systems must teach not only “*how to reach the target”* but also how to maintain stable, low-risk trajectories within strict spatial constraints. Such instructional demands motivate novel simulation and assessment frameworks capable of capturing the uncertainty and variability inherent in human performance.

Meanwhile, the field of surgical data science has emerged as a unifying framework for capturing, modeling, and utilizing the rich multimodal data streams generated in the operating room. Modern machine learning tools and large-scale frameworks, such as TensorFlow, make it feasible to train complex models on kinematic, imaging, and biosignal data. However, deploying these systems in medicine is increasingly constrained by the need for transparency, trust, and explainability. Holzinger et al. (13) have argued that medical Artificial Intelligence (AI) must evolve beyond black-box predictions toward systems that can be scrutinized, justified, and causally interpreted. In the context of neurosurgical training, this implies that performance assessment should not only be accurate but also decomposable into clinically meaningful and interpretable components.

Classical scoring methods, whether threshold-based or fuzzy, remain limited in handling uncertainty. Fuzzy logic offers a range of partial truth values but still reduces performance to a single membership scale, making it hard to explicitly represent indeterminate or conflicting evidence. Recent advances in neutrosophic and plithogenic theories present a promising alternative. Neutrosophic logic extends fuzzy sets by allowing Truth (*T*), Indeterminacy (*I*), and Falsity (*F*) to coexist as independent, non-overlapping components (14). This approach is especially suitable for modeling transitional states—like trainees who get close to a DBS target but display unstable motor patterns, hesitation, or inconsistent control. In such cases, defining performance simply as “correct” or “incorrect,” or using a single fuzzy dimension, risks producing false positives that could compromise patient safety.

Taken together, these developments highlight a clear methodological gap. VR/XR technologies and neuromodulation techniques have advanced rapidly, and surgical data science has provided a conceptual roadmap for managing high-dimensional intraoperative data. However, there remains no formal framework that (i) explicitly models uncertainty in stereotactic trajectories, (ii) breaks down surgical performance into interpretable components, and (iii) can be used as an explainable scoring system within immersive simulators.

In this work, we address this gap by proposing a theoretical Neutrosophic Explainable AI (XAI) framework for stereotactic neurosurgical simulation. We define neutrosophic membership functions *T(d, s), I(d, s)*, and *F(d, s)* based on spatial deviation *(d)* and motor instability *(s)*, and derive a composite competence score *S(d, s)* that maintains a clear band of indeterminacy between visibly competent and clearly unsafe performance. Using a synthetic dataset that simulates three levels of expertise (expert, indeterminate, and novice), we systematically analyze the statistical properties and geometric structure of the neutrosophic competence manifold. Our aim is to deliver a mathematically rigorous, interpretable proof-of-concept that can later be integrated into real VR/XR stereotactic training systems and extended to multimodal surgical data.

## 2 Materials and methods

### 2.1 Theoretical system architecture

**Figure 1:**
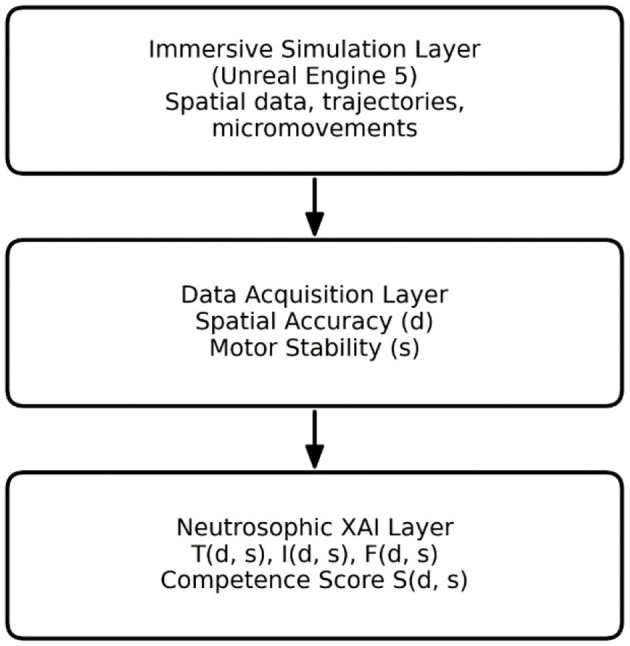
Conceptual system architecture for the Neutrosophic XAI framework.

Given the lack of physical VR hardware and human-subject testing, this study uses a theoretical yet practically consistent architecture to assess stereotactic surgical skills with Neutrosophic Logic. The framework is made up of three conceptual layers (Figure 1).

1. Immersive simulation layer

A high-fidelity virtual environment—conceptually modeled in Unreal Engine 5 (UE5)—is assumed capable of generating kinematic proxies of surgical behavior, including spatial trajectories, micromovements, and optional physiological surrogates. No physical VR implementation was performed; the layer serves as a theoretical generator of the behavioral signals required for evaluation.

2. Data acquisition layer

This layer aggregates variables essential to stereotactic precision:

- Spatial accuracy (Euclidean deviation from the intended anatomical target)
- Motor stability (temporal variance capturing tremor and micromovement amplitude)

These two variables constitute the inputs to the Neutrosophic evaluation process.

3. Neutrosophic explainable AI (N-XAI) layer

Raw performance variables are converted into three separate membership functions—Truth *(T)*, Indeterminacy *(I)*, and Falsity *(F)*—that collectively describe surgical skill, uncertainty, and mistakes. A final competence index *S(d, s)* offers an understandable summary that helps distinguish expert, borderline, and novice behaviors.

### 2.2 Performance variables and kinematic definitions

Two psychomotor metrics were used to characterize stereotactic performance:

#### 2.2.1 Spatial accuracy (d)

Deviation between the instrument tip and the stereotactic target is modeled as:


$$d=\sqrt{{\left(x_{t}-x_{s}\right)}^{2}+{\left(y_{t}-y_{s}\right)}^{2}+{\left(z_{t}-z_{s}\right)}^{2}}$$

where (*xt, yt, zt*) denote the target coordinates and (*xs, ys, zs*) the participant-generated position. Because spatial deviation is inherently non-negative, simulated samples drawn from Gaussian priors were rectified using an absolute-value operator to ensure:


$$\begin{array}{l} d\geq 0 \end{array}$$

#### 2.2.2 Motor stability (s)

Motor instability is modeled as the positional variance of the instrument trajectory:


$$\begin{array}{l} s=Var\left(p\left(t\right)\right),\;p\left(t\right)\in {\mathbb{R}}^{2} \end{array}$$

where p(t) represents the tracked instrument-tip position over time. The resulting scalar *s* has units of mm^2^ and captures the dispersion of micromovements around the intended trajectory, reflecting tremor and motor instability.

In an empirical VR/XR simulator, *s* would be computed directly from kinematic time-series data over a fixed temporal window. In the present proof-of-concept, full trajectories are not simulated; instead, scalar values of *s* are sampled from class-specific Gaussian distributions N (μ_*s*_, σ_s_) as a compact surrogate for the aggregated variance that would arise from such time-series measurements. As variance is inherently non-negative, simulated samples were rectified to ensure *s*≥ 0.

#### 2.2.3 Psychomotor representation

Each virtual participant is represented by the behavioral vector:


$$\begin{array}{l} X=\left(d,\;s\right) \end{array}$$

which serves as input to the Neutrosophic XAI model.

### 2.3 Neutrosophic modeling of surgical competence

Surgical performance is formalized as a Single-Valued Neutrosophic Set (SVNS) (14):


$$\begin{array}{l} A=\langle T\left(X\right),I\left(X\right),F\left(X\right)\rangle ,\;X=\left(d,\;s\right) \end{array}$$

with each membership function satisfying:


$$\begin{array}{l} T,I,F\in \left[0,1\right],\;0\leq T+I+F\leq 3 \end{array}$$

Unlike probabilistic or fuzzy scoring, neutrosophic logic treats competence, uncertainty, and error as independent dimensions, capturing ambiguous states frequently observed in neurosurgical training.

#### 2.3.1 Truth function—competent performance


$$\begin{array}{l} T\left(d,\;s\right)=\max\left(0,\;1-\frac{d}{d_{\max}}\right)•max\left(0,\;1-\frac{s}{s_{\max}}\right) \end{array}$$

where *s*_*max*_ = 3 mm and *s*_*max*_= 0.30 denotes the upper accepted limit of motor instability used for normalization.

#### 2.3.2 Falsity function—performance error

Falsity measures the degree to which performance is clinically unsafe. Distance- and instability-related components are defined as:


$$\begin{array}{l} F_{d}\left(d\right)=\min\left(1,\;\frac{d}{d_{crit}}\right),\;F_{s}\left(s\right)=\min\left(1,\;\frac{s}{s_{\max}}\right) \end{array}$$

where *d*_*crit*_ = 4 mm, marks the threshold for unacceptable deviation.

Overall falsity is determined by:


$$\begin{array}{l} F\left(d,s\right)=\max\left(F_{d}\left(d\right){,\;F}_{s}\left(s\right)\right) \end{array}$$

reflecting a safety-critical “worst-case” criterion.

#### 2.3.3 Indeterminacy function—hesitation/risk zone

Indeterminacy describes borderline performance states where the trainee is neither a clear expert nor an obviously unsafe novice. In stereotactic neurosurgery, this refers to cases where the final spatial deviation is near clinical tolerance, but motor instability is still significant.

To model this behavior, we define a triangular distance kernel centered around a mid–risk distance and adjust it using the normalized motor instability.


$$\begin{array}{l} I\left(d,\;s\right)=\max\left(0,\;1-|\frac{d-d_{mid}}{d_{range}}|\right)•min\left(1,\frac{s}{s_{\max}}\right) \end{array}$$

where *d*_*mid*_ = 1.5 mm indicates the boundary between precise and risky targeting, *d*_*range*_ = 1.5 mm specifies the width of the ambiguous zone, and *s*_*max*_ = 0.30 is the maximum acceptable level of motor instability used for normalization. The triangular component reaches its peak near *d*mid and decreases linearly to zero outside the mid–risk zone, while the factor *s/s*max enhances indeterminacy when the trainee shows higher instability. Therefore, a participant who hits the target distance but exhibits noticeable tremor will have high *I(d, s)*, setting them apart from genuine experts with steady trajectories.

#### 2.3.4 Model parameterization and clinical rationale

The numerical values assigned to the model parameters (*s*_*max*_, *s*_*max*_, *d*_*crit*_, *d*_*mid*_, *d*_*range*_) were selected based on commonly reported accuracy constraints in stereotactic neurosurgery, particularly in deep brain stimulation (DBS) procedures.

Clinical evidence consistently emphasizes that accurate placement of DBS electrodes is critical for therapeutic efficacy. Systematic reviews and large clinical series report that targeting deviations within approximately 2 mm are generally considered acceptable, while even misplacements of this magnitude may already begin to affect clinical outcomes due to the small size and steep functional gradients of subcortical targets such as the subthalamic nucleus (15, 16).

Accordingly, *d*_*mid*_ was set to 1.5 mm to represent the transition between high-precision targeting and the onset of increased clinical risk, while *d*_*range*_= 1.5 mm defines a clinically meaningful uncertainty band spanning the commonly reported tolerance interval (≈1.5–3.0 mm). The parameter *s*_*max*_ = 3 mm reflects the upper bound of generally accepted targeting error, and *d*_*crit*_ = 4 mm marks a deviation level beyond which performance is considered unsafe (17, 18). The instability normalization parameter *s*_*max*_ was set to 0.30 mm to reflect the upper bounds of tremor or micromovement variance reported in psychomotor analyses of high-precision surgical tasks. This value is motivated by high-precision recordings of instrument-tip motion in authentic microsurgical settings, which have estimated the RMS amplitude of physiological tremor to be approximately 0.182 mm. By selecting *s*_*max*_ = 0.30 mm, the model establishes a conservative upper bound that accounts for baseline physiological tremor while providing sufficient headroom for supra-physiological instability—such as hand-drifting, fatigue, or novice-level tremors—within the proof-of-concept framework (19).

Importantly, these numerical values are intended as illustrative defaults for validation and do not represent fixed clinical thresholds. All parameters are explicitly defined in the open-access Python implementation and can be adjusted based on expert judgment, procedure-specific safety margins, or empirical calibration using real surgical or simulator-derived data, without altering the underlying neutrosophic logic or the interpretability of the model. This design ensures that the proposed framework remains flexible, transparent, and adaptable to different neurosurgical procedures, institutional protocols, and evolving clinical standards.

#### 2.3.5 Neutrosophic competence score

The competence score *S* is computed as:


$$\begin{array}{l} S\left(d,s\right)=\frac{2+T\left(d,s\right)-I\left(d,s\right)-F\left(d,s\right)}{3} \end{array}$$

follows the class of score functions commonly defined for Single-Valued Neutrosophic Sets, where Truth increases the score while Indeterminacy and Falsity reduce it, yielding a bounded and order-preserving measure suitable for decision and ranking tasks (20). In the present context, this formulation explicitly penalizes both active error and unresolved uncertainty, reflecting that in high-stakes neurosurgical tasks hesitation and error equally detract from clinical competence, while its linear structure preserves transparency and interpretability in line with the objectives of explainable AI.

### 2.4 Synthetic data simulation

#### 2.4.1 Generative distributions for spatial accuracy and motor stability

To assess the model under controlled and fully reproducible conditions, a synthetic dataset was created to simulate three typical psychomotor profiles observed in stereotactic neurosurgery: experts, indeterminate performers, and novices. Each virtual participant is described by the vector


$$\begin{array}{l} X=\left(d,s\right) \end{array}$$

where *d* represents spatial deviation and *s* denotes motor instability quantified as an aggregated positional variance.

Class-specific Gaussian priors were defined as:

- Experts (*n* = 20)

$$\begin{array}{l} d\sim \;N\;\left(0.5,\;0.20\right),\;s\;\sim \;N\;\left(0.05,\;0.01\right) \end{array}$$

- Indeterminate (*n* = 20)

$$\begin{array}{l} d\sim \;N\;\left(2.0,\;0.30\right),\;s\;\sim \;N\;\left(0.15,\;0.05\right) \end{array}$$

- Novices (*n* = 20)

$$\begin{array}{l} d\sim \;N\;\left(4.0,\;0.50\right),\;s\;\sim \;N\;\left(0.25,\;0.07\right) \end{array}$$

In all cases, *N (*μ, σ*)* denotes a normal distribution with mean μ and standard deviation σ describing inter-subject variability of the aggregated instability measure, rather than temporal variance of individual trajectories. Although *s* is formally defined as a variance-based quantity (Section 2.2.2), here it is treated as a scalar surrogate representing the dispersion of micromovements at the participant level.

Since both variables represent physically non-negative quantities, simulated samples were rectified using an absolute-value operator:


$$\begin{array}{l} d=\max\;\left(0,\;d\right),\;s=max\left(0,s\right) \end{array}$$

ensuring *d* ≥ 0 and *s* ≥ 0 while preserving the statistical properties of each Gaussian prior.

#### 2.4.2 Neutrosophic evaluation pipeline

For each simulated participant:

1. Compute the membership functions *T(d, s), I(d, s)*, and *F(d, s)*.
2. Compute the competence score *S(d, s)*.
3. Assemble all variables into a structured dataset:
4. {*d, s, T, I, F, S*, class label}.

#### 2.4.3 Visualization and interpretation

Scatter plots, membership-function profiles, and competence-surface mappings were created to demonstrate class separability and the geometric characteristics of the neutrosophic model.

#### 2.4.4 Sample size justification

Simulating *n* = 20 samples per class provides sufficient distributional variability for:

- Estimating membership-function behaviors,
- Producing stable non-parametric statistics (Kruskal–Wallis and Dunn tests),
- Visualizing competence landscapes *S(d, s)*,
- Avoiding excessive granularity or overfitting.

This follows established practices in synthetic modeling for surgical simulation research.

### 2.5 Computational implementation

All computations were performed in Python 3.1 using NumPy, pandas, Matplotlib, SciPy, and scikit-posthocs, along with custom functions implementing the neutrosophic membership model. The complete computational pipeline—including data generation, evaluation of *T(d, s), I(d, s)*, and *F(d, s)*, statistical analyses, and figure creation—is fully reproducible using the public script neutrosophic_xai_stereotactic_neurosurgical_simulation.py archived in the associated GitHub/Zenodo repository.s fully reproducible using the public script neutrosophic_xai_stereotactic_neurosurgical_simulation.py archived in the associated GitHub/Zenodo repository. Analyses were performed using Python 3.11 (Python Software Foundation, Wilmington, DE, USA).

All intermediate and final outputs generated by this pipeline, including (i) the synthetic dataset, (ii) descriptive and inferential statistics, and (iii) figure-ready matrices for visualization, are included as Supplementary materials.

- Supplementary Dataset S1: synthetic neutrosophic dataset (CSV)
- Supplementary Table S2: statistical outputs [Shapiro–Wilk (21), Kruskal–Wallis (22), Dunn *post-hoc* (23)].
- Supplementary File S3: complete reproducible Python code.

Together, these materials ensure full transparency and end-to-end reproducibility of all numerical and graphical results reported in this manuscript.

### 2.6 Statistical analysis

Normality of competence scores was assessed using the Shapiro–Wilk test. As normality was violated in at least one group, non-parametric Kruskal–Wallis *H* tests were applied, followed by Dunn's *post-hoc* pairwise comparisons with Bonferroni correction (23). Statistical significance was set at: α = 0.05.

## 3 Results

### 3.1 Simulation outcomes and group-level differences

A total of 60 synthetic surgical attempts were performed across three expertise levels (expert, indeterminate, and novice). Neutrosophic competence scores *S(d, s)* demonstrated clear stratification among the groups (Table 1).

**Table 1:** Descriptive statistics of neutrosophic competence scores across simulated trainee groups.

**Group**	***n***	**Mean ±SD**	**Median**	**Min–Max**
Expert	20	0.832 ± 0.033	0.838	0.775–0.898
Indeterminate	20	0.420 ± 0.085	0.437	0.257–0.536
Novice	20	0.343 ± 0.013	0.333	0.333–0.367

The Indeterminate group showed the greatest variation, aligning with its middle position in the psychomotor spectrum.

**Figure 2:**
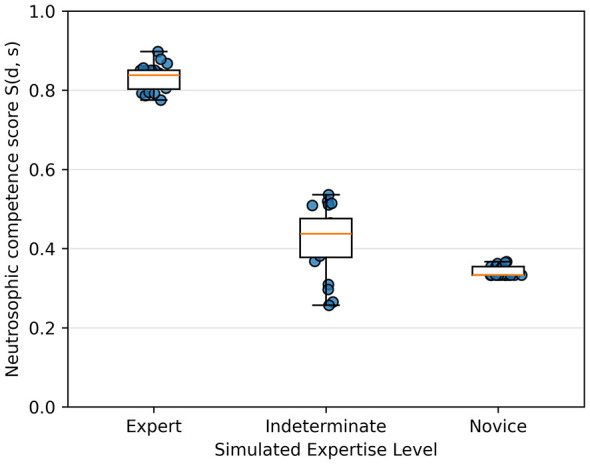
Boxplots of neutrosophic competence scores (??, ??) across groups.

Normality was evaluated using the Shapiro–Wilk test (Table 2a). The Novice group violated normality assumptions (*p* = 0.0001), while the Expert and Indeterminate groups did not. As a result, group comparisons were performed with a Kruskal–Wallis test, which showed a highly significant effect of expertise level on *S (d, s) (H* = 44.42, *p* = 2.26 × 10^−10^; Table 2b). Boxplots in Figure 2 visually confirm the clear separation between groups.

**Table 2:** Statistical analysis of group differences in neutrosophic competence scores.

**(a) Shapiro–Wilk normality**			
**Group**	***W***	***p*** **-Value**	**Normality**
Expert	0.9643	0.6327	Yes
Indeterminate	0.9244	0.1203	Yes
Novice	0.7274	0.0001	No
**(b) Kruskal–Wallis omnibus test**			
**Test**	***H*** **-value**	***p*** **-Value**	**Interpretation**
Kruskal–Wallis	44.4186	2.26 × 10^−10^	Groups differ significantly
**(c) Dunn** ***post-hoc*** **comparisons (Bonferroni-corrected)**			
**Comparison**		***p*** **-Value**	**Interpretation**
Expert vs. Indeterminate		3.8 × 10^−5^	Significant
Expert vs. Novice		<10^−6^	Significant
Indeterminate vs. Novice		0.087430	Not significant

*Post-hoc* Dunn tests with Bonferroni correction revealed statistically significant pairwise differences between Experts and both Indeterminate and Novice groups, while the Indeterminate–Novice comparison did not reach significance after adjustment (Table 2c), suggesting some overlap among mid-range performers.

*Post-hoc* pairwise comparisons using Dunn's test revealed that Experts were significantly differentiated from both other groups (*p* < 0.001). In contrast, the difference between Indeterminate and Novice performers did not reach statistical significance (*p* = 0.087430). This pattern reflects the presence of an intermediate performance region, in which borderline and novice behaviors partially overlap, rather than a strictly binary separation. Figure 2 summarizes these results, displaying the competence score distributions. While the Expert and Novice groups show distinct separation, the Indeterminate group intentionally spans an intermediate range, capturing the transition between skill levels.

These results confirm that the neutrosophic scoring model generates statistically reliable and distinct competence profiles for the Expert and Novice groups, while intentionally maintaining an intermediate zone of uncertainty.

### 3.2 Classical vs. neutrosophic evaluation models

**Figure 3:**
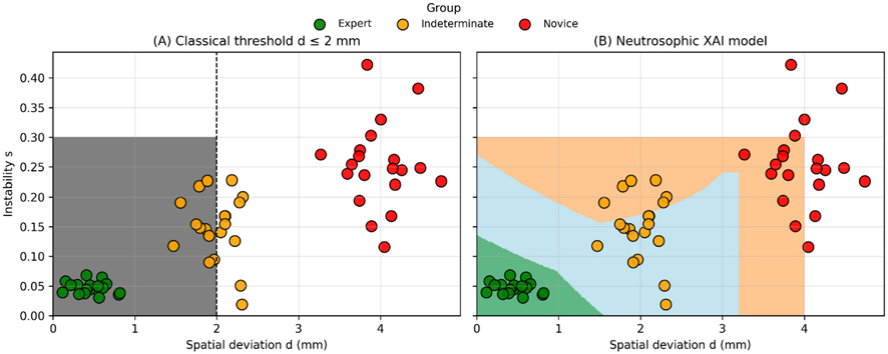
Comparison between classical threshold-based assessment and the proposed neutrosophic XAI model. **(A)** Classical threshold model using a fixed spatial deviation criterion (d = 2 mm), which classifies cases solely based on distance to target. **(B)** Decision regions generated by the neutrosophic XAI model in the distance–instability space, showing how competence is determined by the combined effect of spatial deviation (d) and motor instability (s).

Classical competency evaluation that relies solely on geometric deviation classifies trajectories with *d* ≤ 2 mm as “pass.” As shown in Figure 3A, several Indeterminate attempts would be labeled as competent under this criterion despite showing elevated instability *s*. In contrast, the neutrosophic model evaluates deviation and instability together through the membership functions *T(d, s), I(d, s)*, and *F(d, s)*. In Figure 3B, attempts with acceptable deviation but reduced stability fall into intermediate or low competence categories *S(d, s)* ≈ 0.35–0.55), preventing false-positive classifications.

### 3.3 Visual interpretation of competence clusters

**Figure 4:**
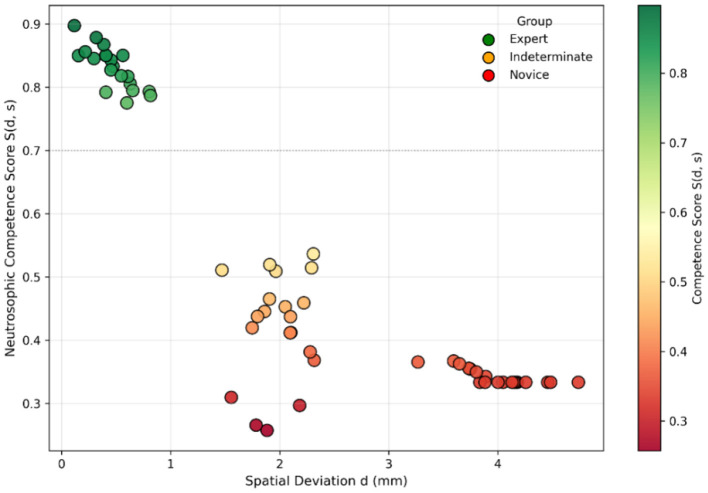
Scatter plot of synthetic trainee performance mapped to *S(d, s)*.

Figure 4 displays all samples in the *(d, s)* plane, colored by their neutrosophic score S(d, s). Three coherent clusters appear:

- Experts: near-zero deviation and minimal instability, with high scores.
- Indeterminate performers: intermediate deviation with heterogeneous instability and intermediate scores.
- Novices: large deviation and high instability, with low scores.

The area around *d* ≈ 1.5–2.0 mm includes both Indeterminate and some Novice samples, indicating that spatial deviation alone cannot reliably distinguish borderline from unsafe performance and supporting the combined use of *d* and *s*.

### 3.4 Behavior of neutrosophic membership functions

**Figure 5:**
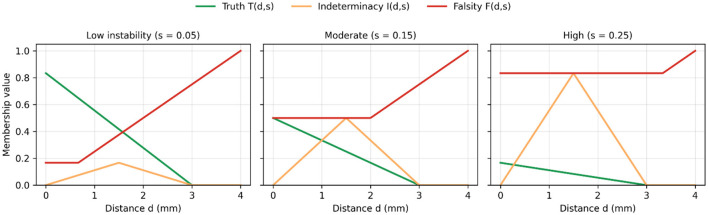
Neutrosophic membership functions for three instability conditions.

Figure 5 shows the neutrosophic membership functions across three instability levels (*s* = 0.05, 0.15, 0.25). For all conditions, Truth *T(d, s)* decreases consistently with distance, while Falsity *F(d, s)* increases toward clinically unsafe areas. Indeterminacy *I(d, s)* peaks in the mid-range interval (*d* ≈ 1.5–2.0 mm).

At low instability (*s* = 0.05), indeterminacy stays low, and most of the range is dominated by either truth or falsity. At moderate instability (*s* = 0.15), a clear band of indeterminacy appears around the mid-risk distances. Under high instability (*s* = 0.25), falsity takes over most of the domain, and truth remains near zero, even for relatively small deviations.

### 3.5 Geometry of the neutrosophic competence surface

**Figure 6:**
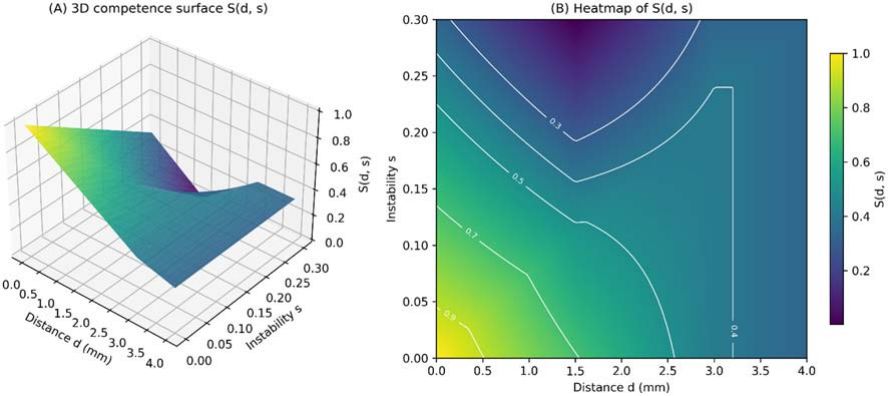
Visualization of the neutrosophic competence function *S(d, s)*. **(A)** Three-dimensional surface representation of the competence score as a function of spatial deviation (d) and instability (s). **(B)** Two-dimensional heatmap of *S(d, s)* with contour lines indicating score gradients across the distance–instability space.

The overall behavior of *S(d, s)* is summarized in the combined surface–heatmap display in Figure 6. Panel A presents a 3D competence surface, where a high-performance plateau occurs at low deviation and low instability, followed by a gradual curve toward medium and low scores as either variable increases. Panel B shows the corresponding heatmap with contour lines at *S* = 0.3, 0.5, and 0.7, which define expert, indeterminate, and unsafe zones. These visual representations emphasize the non-linear interaction between *d* and *s*, especially in the mid-risk area around *d* ≈ 1.5–2.0 mm, where slight changes in instability can move a trajectory from expert-like to indeterminate or unsafe competence levels.

## 4 Discussion

### 4.1 Conceptual implications of the neutrosophic XAI framework

The results show that the neutrosophic framework captures aspects of surgical performance that other evaluators cannot access. As shown in Figure 3A, relying only on geometric criteria (e.g., *d* ≤ 2 mm) incorrectly classifies many unstable trajectories from the Indeterminate group as competent—an issue often seen in VR neurosurgical training (3, 5).

In contrast, the neutrosophic formulation incorporates deviation and instability through independent membership functions *T(d, s), I(d, s)*, and *F(d, s)*. This allows for competence scoring that reflects execution quality instead of just spatial accuracy, addressing a limitation noted in cognitive–motor studies (24).

This conceptual advantage is clear in Figure 2, where the score distributions of Expert and Novice groups do not overlap—an effect that single-axis metrics can't reproduce. By allowing Truth (competence), Indeterminacy (hesitation or borderline risk), and Falsity (unsafe behavior) to coexist, the framework matches modern views of surgical performance as a multidimensional cognitive–motor process. Additionally, the model's interpretability aligns with ongoing efforts to incorporate explainability into medical AI systems (25).

### 4.2 Statistical properties of the neutrosophic score

The statistical results in Table 2 and Figure 2 illustrate the internal mathematical structure of the competence score *S(d, s)*.

The Novice group violated normality (*W* = 0.727, *p* = 0.0001), a result expected because novice behavior leads to nearly deterministic membership outcomes (*T* → 0, *I* → 0, *F* → 1), resulting in a truncated distribution centered around *S* ≈ 0.333. This behavior aligns with the properties of the Shapiro–Wilk normality test (21).

Expert and Indeterminate groups showed wider distributions, indicating real variation in the combinations of *T, I*, and *F* produced by their psychomotor patterns.

Dunn *post-hoc* contrasts were highly significant for Expert–Novice and Expert–Indeterminate comparisons (*p* < 10?5), aligning with the separation of clusters in Figure 4. These results collectively confirm the discriminative ability and numerical stability of the neutrosophic metric across different levels of expertise.

### 4.3 Geometry of the neutrosophic competence manifold

The geometric structure of *S(d, s)*, summarized in Figure 6, reveals clinically meaningful regions:

- A high-performance plateau where Truth prevails (low deviation, low instability), which directly aligns with the Expert cluster in Figure 4.
- An uncertainty valley—visible as a curved depression in the surface plot and as the band around the 0.4–0.6 contours in the heatmap—corresponds with the Indeterminate group's spread in Figure 4 and the peak of the Indeterminacy function in Figure 5. This region aligns with psychomotor transitional states described in neurosurgical training literature (7, 26).
- A low-competence collapse region, where performance becomes unsafe as deviation or instability rises, mirroring the lower-left area in Figure 6B.

The heatmap also displays highly non-linear decision boundaries, especially around *d* ≈ 1.5–2.0 mm, where small increases in instability cause sudden shifts in competence classifications. This behavior reflects real clinical constraints: in DBS, even millimeter deviations or loss of stability can greatly impact outcomes (8, 9, 27).

Thus, the manifold geometry confirms that the neutrosophic formulation offers a mathematically interpretable and clinically relevant model of skill assessment.

### 4.4 Indeterminacy as a missing construct in surgical assessment

A key contribution of this work is the explicit modeling of Indeterminacy, a dimension missing from traditional evaluation systems. Figure 5 shows that the *I(d, s)* function peaks at moderate deviations (1.5–2.0 mm) and increases with greater instability. This pattern directly relates to the spread and variability of the Indeterminate cluster in Figure 4 and its high variance in Table 1. Such transitional psychomotor states—where trainees reach spatial targets but do so inconsistently or with tremor—are frequently reported in surgical skill acquisition research. Classical binary evaluators label these attempts as “*almost correct”*; however, the neutrosophic framework appropriately situates them within an explicit uncertainty zone, thereby preventing premature advancement and enhancing the overall safety and validity of competency assessment.

### 4.5 Advantages over classical and fuzzy-logic models

Figures 3–6 collectively demonstrate the limitations of classical and fuzzy approaches:

- Classical thresholding collapses multidimensional psychomotor behavior into binary decisions, masking risk-laden but spatially accurate attempts (3).
- Fuzzy logic improves granularity but lacks the capacity to model uncertainty independently (14).

The neutrosophic model:

- Assigns low competence to superficially accurate but unstable attempts (Figures 3B, 4).
- Distinguishes borderline from expert-level performance (Figure 5).
- Produces smooth and clinically interpretable competence landscapes (Figure 6).

These advantages are especially relevant for high-precision procedures such as DBS, where execution stability is as critical as spatial accuracy (8, 9).

### 4.6 Implications for explainable AI in VR/XR surgical education

Because Truth, Indeterminacy, and Falsity are calculated independently, each trainee attempt provides a clear explanation of performance classification. This aligns with modern XAI principles in medicine (25).

The framework is compatible with next-generation XR surgical systems that incorporate:

- Tremor spectra
- Gaze tracking
- Force profiles
- Physiological workload indices

and aligns with ongoing developments in digital surgery and real-time surgical data science (2, 4, 28, 29).

Thus, the neutrosophic model addresses a methodological gap where assessment tools fall behind the sophistication of modern immersive simulators.

### 4.7 Limitations

The present study has several limitations that should be acknowledged when interpreting its results. First, the proposed framework is evaluated using synthetic data, designed to emulate representative psychomotor profiles rather than being derived from empirical VR/XR simulator recordings or intraoperative measurements. As such, the reported separability between performance groups should be interpreted as a demonstration of the internal consistency and explainability of the neutrosophic model, rather than as evidence of real-world predictive accuracy. Empirical validation using simulator-based or clinical datasets constitutes an essential next step.

Second, the framework relies on a set of explicitly defined model parameters (e.g., dmax, dcritd, smax) that are presented as clinically motivated but illustrative defaults. Although these parameters were selected based on psychomotor literature and conservative safety considerations, no formal sensitivity analysis is performed in the current work. Consequently, parameter calibration and robustness should be systematically explored in future studies using procedure-specific data, expert input, and statistical validation.

Third, the current implementation intentionally adopts a minimal model scope, operating on only two variables—spatial deviation and motor instability—to preserve interpretability and analytical transparency in an Explainable AI context. While this reduced dimensionality facilitates theoretical analysis and pedagogical clarity, it does not capture the full multimodal complexity of neurosurgical skill, which may involve additional kinematic, force, gaze, temporal, and physiological features. .Future extensions of the framework will address this limitation by integrating richer data modalities; notably, the neutrosophic structure is inherently scalable, allowing for the addition of new “Truth, Indeterminacy, and Falsity” dimensions for each new variable without requiring a complete redesign of the scoring logic.

## 5 Conclusions

This study introduces the first Neutrosophic XAI framework for assessing competence in stereotactic surgical simulation. By analyzing performance into Truth, Indeterminacy, and Falsity, the model identifies transitional psychomotor states that traditional and fuzzy systems often miss.

Synthetic experiments show that the framework:

- Discriminates expertise levels with strong statistical resolution (Figure 2, Table 2).
- Identifies unstable yet superficially accurate attempts, preventing false-positive competency assignments (Figures 3B, 4).
- Reveals a clinically meaningful uncertainty band, represented by the Indeterminacy peak and the mid-risk valley in the competence manifold (Figures 5, 6).
- Offers transparent, interpretable reasoning, making it suitable for integration into VR/XR surgical education systems.

These findings establish a solid foundation for future empirical validation and pave the way for next-generation immersive simulators where competence assessment is mathematically grounded, uncertainty-aware, and explainable.

## Data Availability

The datasets presented in this study can be found in online repositories. The names of the repository/repositories and accession number(s) can be found at: https://doi.org/10.5281/zenodo.17905071.

## Nomenclature

### Resource Identification Initiative (RRID)

This study does not involve biological reagents, antibodies, cell lines, organisms, software packages with RRID registration, or other experimental resources typically covered by the Resource Identification Initiative. All computational components are fully detailed in the Materials and Methods section, and the complete Python code used to generate figures, tables, and statistical analyses is publicly available in the GitHub/Zenodo repository associated with this manuscript. If future versions of the simulation framework include RRID-registered software or hardware components (such as VR tracking systems or biosignal acquisition devices), the relevant catalog number and RRID will be provided in accordance with the Resource Identification Initiative guidelines.

### Life Science Identifiers (LSIDs)

This manuscript does not involve zoological nomenclatural acts, new species descriptions, taxonomic revisions, or any activity requiring Zoobank registration. Therefore, no Life Science Identifiers (LSIDs) are applicable. Because the present study is purely computational and theoretical, no LSIDs are required or included. Additional Requirements.
